# Differentiation of Glioma and Radiation Injury in Rats Using *In Vitro* Produce Magnetically Labeled Cytotoxic T-Cells and MRI

**DOI:** 10.1371/journal.pone.0009365

**Published:** 2010-02-26

**Authors:** Ali S. Arbab, Branislava Janic, Kourosh Jafari-Khouzani, A. S. M. Iskander, Sanath Kumar, Nadimpalli R. S. Varma, Robert A. Knight, Hamid Soltanian-Zadeh, Stephen L. Brown, Joseph A. Frank

**Affiliations:** 1 Cellular and Molecular Imaging Laboratory, Department of Radiology, Henry Ford Hospital, Detroit, Michigan, United States of America; 2 Department of Neurology, Henry Ford Hospital, Detroit, Michigan, United States of America; 3 Department of Radiation Oncology, Henry Ford Hospital, Detroit, Michigan, United States of America; 4 Frank Laboratory, Radiology and Imaging Sciences, Clinical Center, National Institutes of Health, Bethesda, Maryland, United States of America; 5 Frank Laboratory, National Institute of Biomedical Imaging and Bioengineering, National Institutes of Health, Bethesda, Maryland, United States of America; The University of Chicago, United States of America

## Abstract

**Background:**

A limitation with current imaging strategies of recurrent glioma undergoing radiotherapy is that tumor and radiation injury cannot be differentiated with post contrast CT or MRI, or with PET or other more complex parametric analyses of MRI data. We propose to address the imaging limitation building on emerging evidence indicating that effective therapy for recurrent glioma can be attained by sensitized T-cells following vaccination of primed dendritic cells (DCs). The purpose of this study was to determine whether cord blood T-cells can be sensitized against glioma cells (U-251) and if these sensitized cytotoxic T-cells (CTLs) can be used as cellular magnetic resonance imaging probes to identify and differentiate glioma from radiation necrosis in rodent models.

**Methodology/Principal Findings:**

Cord blood T and CD14+ cells were collected. Isolated CD14+ cells were then converted to dendritic cells (DCs), primed with glioma cell lysate and used to sensitize T-cells. Phenotypical expression of the generated DCs were analyzed to determine the expression level of CD14, CD86, CD83 and HLA-DR. Cells positive for CD25, CD4, CD8 were determined in generated CTLs. Specificity of cytotoxicity of the generated CTLs was also determined by lactate dehydrogenase (LDH) release assay. Secondary proliferation capacity of magnetically labeled and unlabeled CTLs was also determined. Generated CTLs were magnetically labeled and intravenously injected into glioma bearing animals that underwent MRI on days 3 and 7 post- injection. CTLs were also administered to animals with focal radiation injury to determine whether these CTLs accumulated non-specifically to the injury sites. Multi-echo T2- and T2*-weighted images were acquired and R2 and R2* maps created. Our method produced functional, sensitized CTLs that specifically induced U251 cell death *in vitro*. Both labeled and unlabeled CTLs proliferated equally after the secondary stimulation. There were significantly higher CD25 positive cells (p = <0.006) in CTLs. In addition, T2- and T2*-weighted MR images showed increased low signal intensity areas in animals that received labeled CTLs as compared to the images from animals that received control cells. Histological analysis confirmed the presence of iron positive cells in sites corresponding to MRI low signal intensity regions. Significant differences (p = <0.001) in tumor R2 and R2* values were observed among the groups of animals. Animals with radiation injury exhibited neither MRI hypointense areas nor presence of iron positive cells.

**Conclusion:**

Our results indicate that T-cells can be effectively sensitized by *in vitro* methods and used as cellular probes to identify and differentiate glioma from radiation necrosis.

## Introduction

Malignant glioma is one of the most aggressive tumors with a poor prognosis despite the available treatments [1]. Standard treatment procedures, consisting of surgery and radiation therapy (followed by adjuvant chemotherapy), very often fail due to the inability to accurately delineate tumor margins [2]–[4], and the median survival time for patients with recurrent glioblastoma multiforme (GBM) is less than 1 year [5]. The infiltrative nature of GBM is considered to be one of the main factors impeding the complete removal of tumor mass by surgical procedure [6]. Following radiation therapy or surgery, recurrence is common and almost invariably occurs within <2 cm of the prior resection line. Detection of recurring tumor at an early stage using current *in vivo* imaging techniques is difficult, mainly due to normal tissue damage that occurs following radiation or surgery [7], [8]. Hentschel and Sawaya emphasized the need for high quality imaging to detect recurring tumors, indicating that residual or satellite tumor cells have a potential of becoming even more aggressive and resistant to therapy, as compared to the original primary tumor [6].

Unlike the surrounding normal cerebral vasculature, tumor vessels are typically more permeable to contrast agents and can thus be detected by contrast-enhanced magnetic resonance imaging (MRI) or computed tomography (CT). However, areas of radiation injury can also show enhancement due to active inflammation accompanied by an increase in vascular permeability. Differentiating recurrent glioma from radiation injury based only on changes in vascular permeability and/or blood volume based on contrast enhanced MRI or CT is problematic. MR spectroscopy (MRS), diffusion weighted imaging (DWI) and mapping of the apparent diffusion coefficient (ADC) have produced mixed results in differentiating recurrent tumor from radiation injury [9], [10]. It has been reported that MRS and ADC values, alone or combined, are not conclusive in discriminating between tumor recurrence and radiation injury when an admixture of microscopic tumor and necrotic tissues are present in the brain [9]. In addition, localization of observed MRS changes requires co-registration of MRS data with yet another high resolution MRI.

Nuclear medicine imaging techniques such as ^18^F-FDG positron emission tomography (PET) and single photon emission computerized tomography (SPECT) have been used to differentiate recurrent glioma from radiation injury; however, the results have been controversial and inconclusive [11], [12]. PET and SPECT have limited spatial resolution and relatively high cortical background activity, therefore, ^18^F-FDG-PET cannot accurately delineate residual tumor after therapy [13], [14]. Moreover, ^18^F-FDG-PET images also need co-registration with MRI or CT images to differentiate small or suspicious lesions. In contrast, ^11^C-MET-PET is better suited for monitoring the effects of radiation therapy where injury is displayed as a reduction of the relative methionine-uptake. Nonetheless, the short half-life of ^11^C is still considered a significant limitation to the widespread use of this technique [14].

Tumor immunology has long been a focus of cell-based vaccine therapy research. Dendritic, as well as T-cells, are considered to be the best candidates for developing cellular therapies to treat malignancies. Dendritic cell-based vaccination therapy against recurrent glioma that utilizes the patient's own dendritic cells that are pulsed, *ex-vivo*, with the derived glioma cell-lysate is currently in clinical trials [15], [16]. In experimental glioma models, an increase in the number of cytotoxic T-lymphocytes (CTL) compared to control or pre-vaccination levels is observed following the administration of glioma cell-lysate-pulsed dendritic cell therapy. Previous studies by our group have demonstrated initiation of cellular immunity in adoptive transfer model in syngeneic Fisher rats [17]. It has been hypothesized that using a well-established glioma lysate-pulsed dendritic cell technique, sensitized T-cells (i.e. CTLs) can be developed *in vitro*. These CTLs can be magnetically labeled and serve as cellular probes that can be tracked *in vivo* by cellular magnetic resonance imaging (CMRI), to detect and differentiate human glioma from radiation injury.

Recently, we have created superparamagnetic iron oxide (SPIO)-transfection agent complexes for labeling mammalian cells using two FDA approved agents (ferumoxides and protamine sulfate) [18]–[20]. Cells labeled with the ferumoxides-protamine sulfate (**FePro**) complex can be imaged at clinically relevant MRI field strengths using standard imaging techniques, and at the higher fields commonly used for animal experiments. Results from our group and other investigators demonstrated that MRI can track and monitor the migration of magnetically labeled cells in a variety of organs and disease models [21]–[25]. Investigations by our group and other laboratories demonstrate that labeling various types of cells with ferumoxides does not alter the viability and functional capability of these cells, or the differentiation capacity of stem cells [26], [27]. For example, after secondary stimulation with proteolipid protein (PLP), labeled sensitized T-cells exhibited proliferation capacity similar to that of control, non-labeled cells [22]. In summary, cellular MRI acquires high spatial resolution images to detect the accumulation of small numbers of magnetically labeled cells [18], [21]–[23]. This technique may enable the delineation of tumor margins from surrounding tissues in a more precise manner. In addition, identification and precise localization of *in vivo* accumulated cells does not require the combining or registration of additional scans.

The purpose of this study was to determine whether: (1) T-cells collected from cord blood can be sensitized against glioma cells, (2) CTLs can be labeled effectively using ferumoxides-protamine sulfate (FePro) complexes for CMRI, (3) labeled CTLs can be used as probes to identify implanted glioma in a rodent model by CMRI, and whether (4) labeled CTLs can be used to differentiate glioma from radiation necrosis.

## Results

### Production of Mature DC and CTLs Specific for U-251 Tumor Cells

**Figure 1:**
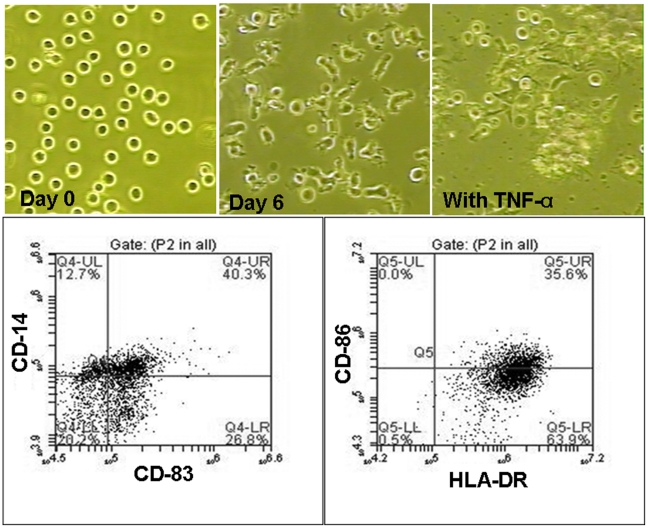
Making of mature dendritic cells from CD14+ cells. **Upper panel:** Morphological changes in CD14+ cells following the addition of immature and mature dendritic cell (DC) media. Cells were grown in RPMI-1640 media containing 10% FBS, IL4 and GCSF; at day 8 TNF-α was also added. CD14+ cell morphology changed from the rounded cells to the cells exhibiting short dendrite-like processes. Mature DC attached to the growth surface. **Lower panel:** Multicolor flow cytometric dot plots of CD14+ cells-derived mature DCs at day 11 (3 days after adding TNF-α) indicate down regulation of CD14 and up regulation of CD86, CD83 and HLA-DR after DCs maturation.

**Figure 2:**
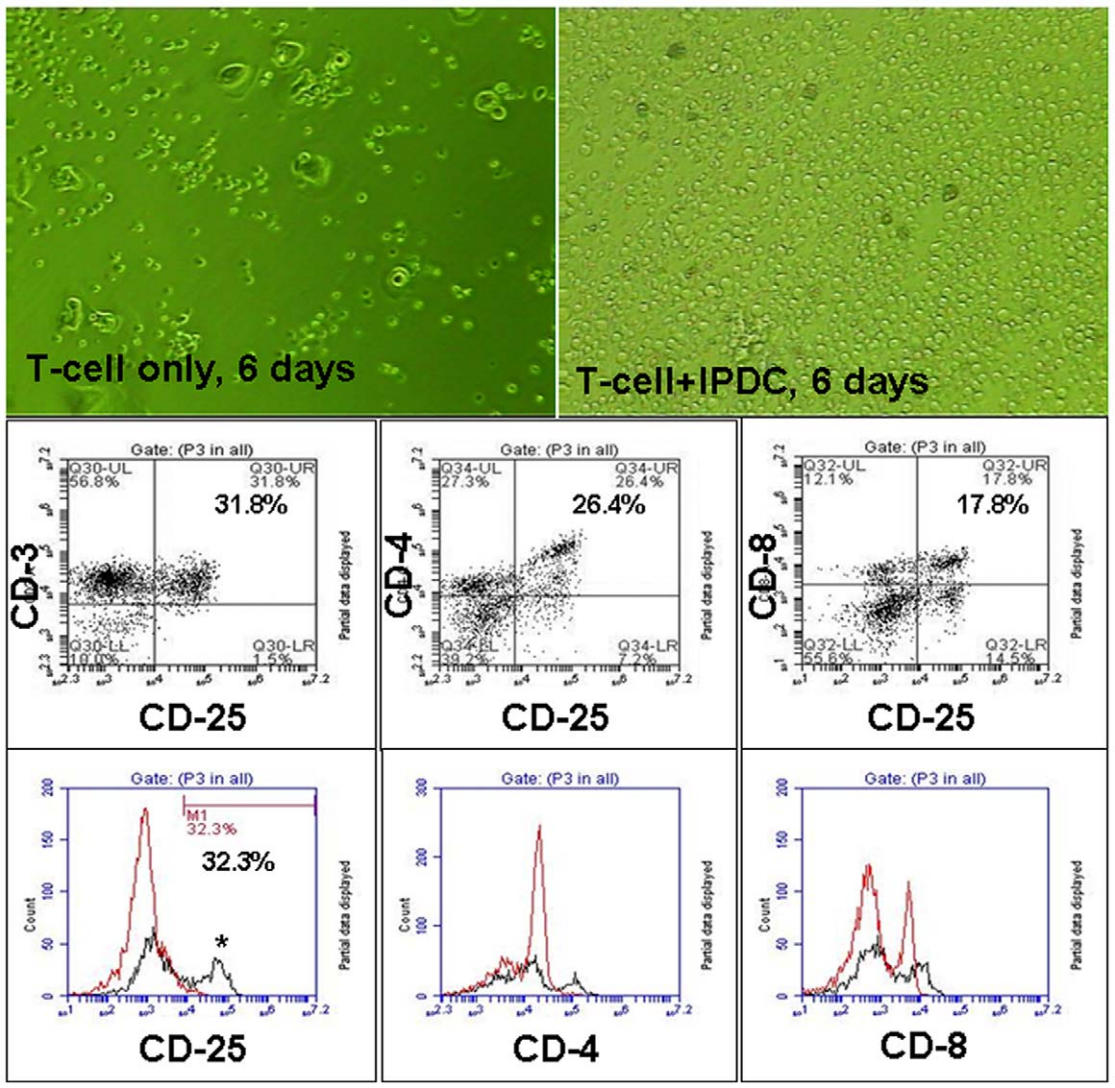
Morphological and phenotypical changes in T-cells during sensitization. **Upper panel:** Culture of cord blood derived T-cells in the absence (left) or presence (right) of U251 cell lysate primed irradiated DC (PIDC). T-cells were cultured in RPMI-1640 media containing 10% FBS and IL-2 for 6 days. Note the number and morphology of the sensitized T-cells (right). **Middle panel:** Dot plot analysis of flow cytometric data of the CTLs showing activated T-cells (CD25) that are also both CD4 and CD8 positive. **Lower panel:** Flow-cytometric analysis (histogram plot) of T-cells cultured in the absence (red line) and presence (black line) of PIDC for day 6. Note the higher number of CD4+, CD8+ and CD25+ T-cells on day 6. There were a significantly (p = 0.006) higher number of activated T-cells on day 6 in the presence of PIDC.

To generate immature and mature DCs, CD14+ cells were isolated from cord blood and first incubated for 4 days in immature DC media that contained GCSF and IL-4. After 4 days of incubation this media, expression of CD14 was down-regulated from 100% to 0.06%, whereas the expression of CD86 immature DC marker was up-regulated (from 56.95±13.82 to 77.95±3.59%). On the other hand, the level of CD83 mature DC marker remained low (1.80±1.23%) (**Supplemental material, Table S1**). At day 5, U-251 tumor cell lysate was added to the generated immature DCs and by day 6, cell started exhibiting morphological changes that were accompanied with the further changes in the expression of DCs' phenotypical markers such as CD83, CD86, HLA-DR (**Figure 1** **,** **Supplemental material, Table S1**). After the addition of TNF-α, that induced DC maturation, an up-regulation of immature CD86 marker (from 77.95±3.59% to 93.44±5.58%) and mature CD83 marker (from 1.80±1.23% to 55.80±3.80%) was observed. Upon maturation, DCs were irradiated and used to sensitize the T-cells. (Supplemental material, Table S1 shows the effect of different doses of cytokines on the expression of CD 14, CD 86, CD 83 and HLA-DR in CD14 derived DCs). Incubation of mature, tumor primed, irradiated DCs with cord blood derived T-cells induced immunological T cell sensitization that resulted in generating CTLs specific for U-251 tumor cells. **Figure 2** shows the morphological as well as the phenotypical changes that were observed through the process of sensitization of T-cells to CTLs, as detected by flow cytometric analysis. T-cells that were co-cultured with irradiated, primed, mature DCs, exhibited higher numbers, as well as the morphological changes such as elongation and increase in size, as compared to control cells. Proliferation assay (**MTT**, (3-(4,5-Dimethylthiazol-2-yl)-2,5-diphenyltetrazolium bromide, a tetrazole) assay) indicated a significant increase in the number of cells that were co-cultured with irradiated primed DCs, as compared to control T cells cultured alone. The optical density (OD) values determined by MTT assay of co-cultured cells were significantly higher (p = <0.05) than combined values of T-cells and irradiated DCs when cultured separately (**Supplemental material, Figure S1**). Flow cytometry detected an increase in the numbers of CD4+, CD8+ and CD25+ (activated T-cells) cells following sensitization, as compared to control T-cells (**Figure 2**). However, significant difference was observed only with CD25 cells (4.57±4.92% vs 31.72±0.13%, p = <0.006).

Next, we determined whether generated CTLs can be labeled effectively using ferumoxides-protamine sulfate (FePro) complexes for CMRI. After 4 h of CTLs incubation with FePro, more than 90% of CTLs were labeled, with the average intracellular iron content of 4.9±0.061 pg/cell. Unlabeled CTLs had an average iron content of 0.113±0.051 pg/cell (p = <0.001). There were no significant differences (p = >0.05) between the labeled and unlabeled CTLs with respect to the viability (55% for unlabeled CTLs and 53% for labeled CTLs) and secondary proliferation capacity when secondarily co-cultured with irradiated primed DCs (OD = 0.26±.003 for unlabeled CTLs and OD = 0.279±.015 for labeled CTLs). (Supplement **Figure S2** shows Prussian blue staining of labeled and unlabeled CTLs).

**Figure 3:**
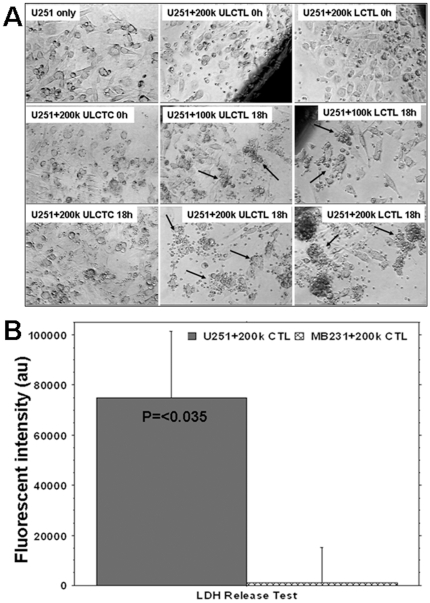
Specificity of labeled and unlabeled cytotoxic T-cells (LCTLs and ULCTLs, respectively) *in vitro*. **Figure 3A:** U251 cells (100k) were plated in 24-well plates and grown until 90% confluent. Then unlabeled control T-cells (ULCTCs), LCTLs or ULCTLs were added to the cultures (100k and 200k per well). The migration and specific accumulation of T-cells around the tumor cells were microphotographed at 0h and 18h of co-culture. **Left column:** Morphology of U251 alone and after incubation with 200k of CTC at 0 and 18 hours. There is no change in the morphology of U251 and CTC seems to be sitting on the tumor cells even after 18 hours of incubation. **Middle column:** Morphology of U251 cells at 0 and 18 hours after incubation with ULCTL. The ULCTLs seem to be sitting on the tumor cells at 0 hour. The specific accumulation of ULCTLs around the tumor cells (arrows) was seen after 18 hours for both 100k and 200k ULCTLs conditions. Compared to the morphology of U251 cells at 0 hour, the dramatic changes in the morphology of U251 cells were observed. Cells become elongated and sparse. This phenomena was more pronounced at higher density (i.e. 200k) of ULCTLs. **Right column:** Similar to ULCTLs, LCTLs also show similar phenomena of specific accumulation around the tumor cells (arrows) and changes in the morphology of U251 cells. **Figure 3B:** Both U251 (100k) and human breast cancer cells (MBA-MD-231, 100k) were incubated in their respective media (1 ml in 24-well plate) in the presence or absence of 200k CTLs overnight. On the next day, supernatants from all the conditions were collected and the lactate dehydrogenase (LDH) contents were determined. Average fluorescent values detected in the supernatant of U251 and MBA-MD-231 cultures without CTLs were deducted from the fluorescent values detected in the corresponding cell lines in the presence of CTLs. The data are expressed as mean ± SEM. Significantly higher (p = <0.017) LDH activity was observed in U251 culture in the presence of CTLs indicating specificity of the sensitized T-cells.

Specificity of CTLs was demonstrated by the 8 fold increase in the number of dead cells in U-251 tumor cells co-cultured with CTLs, compared to that in U-251 tumor cells cultured alone (data not shown). **Figure 3A** shows the morphological changes at 0 and 18 hours of CTL/U-251 cell co-culture and the accumulation of either unlabeled CTLs or labeled CTLs around U251 cells. These changes were not seen when U251 cells were co-cultured with control T-cells. Similar patterns of cell accumulation were observed for both FePro labeled and unlabeled CTLs. U251 cell specific accumulation of CTLs was more prominent in wells that contained 200k of CTLs (Supplement **Figure S3** show CTLs' effect on other glioma cells lines). In addition, significantly (P = <0.035) higher concentrations of LDH were detected in the supernatant of U251 cells co-cultured with CTLs sensitized to U251 compared to that of the supernatant of MBA-MD-231 cells co-cultured with the same CTLs sensitized to U251. These data strongly indicate the immunological specificity of the produced CTLs for U-251 cells (**Figure 3B**). There was increased number of magnetically labeled CTLs observed in subcutaneous gliomas compared to that of breast cancers. The labeled CTLs accumulated not only at the periphery but also in deeper parts of the glioma. Similar accumulation was not observed in breast cancer, which indicates *in vivo* specificity of generated CTLs (**supplement material, Figure S4 (S4A, S4B**)).

### MR Images, Relaxation Maps and Histology –Animal Tumor Model

**Figure 4:**
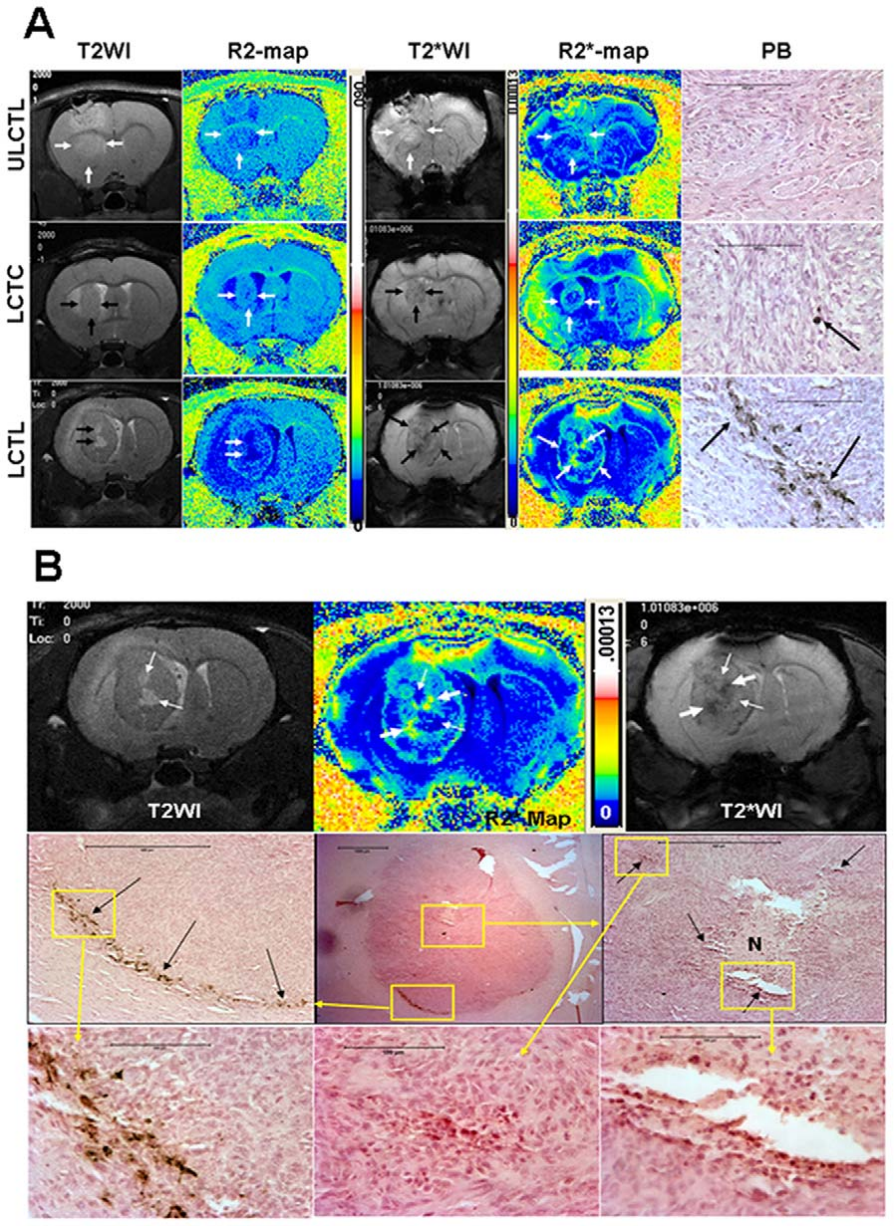
MRI and Prussian blue positive cells in tumors. **Figure 4A:** T2-weighted and T2*-weighted images and their corresponding R2 and R2* maps and DAB enhanced Prussian blue staining from representative animals that received unlabeled CTL (ULCTL, upper row), labeled control T-cells (LCTC, middle row) and CTL (LCTL, lower row). Both T2W and T2*W images show well established tumors in the brain, however, low signal intensity areas were only seen in tumors that received LCTC and LCTL. Corresponding R2* maps show high signal intensity areas. Animals that received LCTL show high signal intensity areas both at the peripheral and central part of the tumors (arrows). Corresponding DAB enhanced Prussian blues staining show multiple Prussian blue positive cells in tumors that received LCTL (arrows). There are a few Prussian blue positive cells seen in tumor that received LCTC (arrow). No definite Prussian blue positive cells were seen in tumor that received ULCTL. Areas of necrosis can easily be identified by comparing T2WI and R2 maps (thick arrows) in tumor that received LCTL (lower row). Bars on the images measure 100 µm. **Figure 4B:** Detailed histological analysis of the tumor that received LCTL. Bars on the images measure 100 µm. **Upper panel:** T2-weighted image (T2WI) and T2*-weighted image (T2*WI) show areas of necrosis (high signal intensity on T2WI and T2*WI and low signal intensity on R2* map). Thin arrows show the sites of necrosis and thick arrows show possible site of accumulated iron positive cells. **Middle panel:** Representative histological section with similar tumor orientation (within the constraints of the experimental limitations, i.e. 1 mm thick MRI slices versus 10 µ thick histological section) show central necrosis (**N**) in the tumor with areas of iron positive cells (black arrows) seen in the central and peripheral part of the tumor that received iron labeled CTLs. **Lower panel:** Enlarged view of the boxed areas.

**Figure 5:**
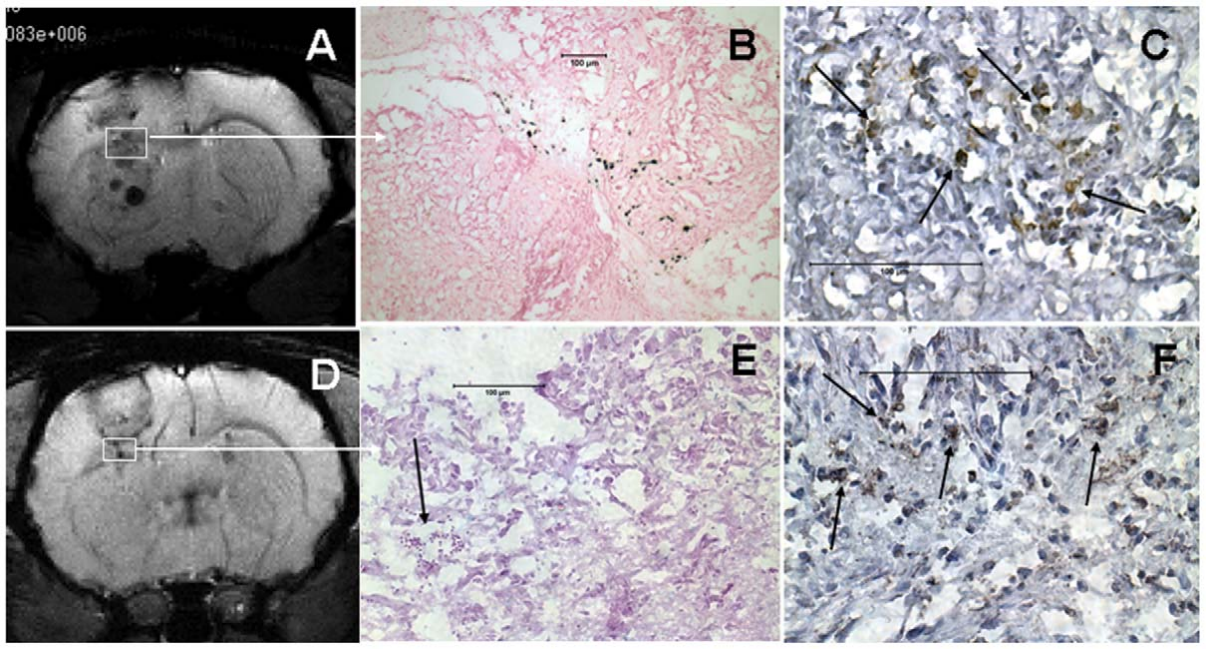
MRI and activated T-cells in tumors. Representative cases of animals that received labeled CTL (A-C) and unlabeled CTL (D-F). (A) T2*-weighted images (T2*WI) showed low signal intensity areas all over the tumors (both periphery and center). (B) DAB enhanced Prussian blue staining showed multiple Prussian blue positive cells corresponding to boxed area (brown spots). (C) Multiple CD45RO positive cells (arrows, activated T-cells) were seen at the corresponding area. (D) T2*WI showed well developed tumor with areas of low signal intensity at the periphery. There were no Prussian blue positive cells seen at the corresponding areas (E), however, multiple hemorrhagic areas were observed (arrow on E). (F) Multiple CD45RO positive cells seen in the tumor. Scale bar  = 100 µm.

T2- and T2*-weighted images (T2WI and T2*WI, respectively) detected growing tumors in the brains of all the animals. Low signal intensity (hypointense) areas were seen near the scalp and within the center of the tumors only in a few animals corresponding to areas of hemorrhage attributed to the tumor cell implantation procedure (i.e. needle track). Corresponding R2 (1/T2) and R2* (1/T2*) maps show high signal intensity areas at the corresponding sites that were different from the high signal intensity seen due to accumulation of Prussian blue positive cells around the periphery of the tumors, (see below). None of the tumors that received unlabeled CTLs showed hypointense regions on T2WI and T2*WI around the tumors, however no Prussian blue positive cells were seen in or around the tumors on histological exams (**Figure 4A** **, upper panel**). In comparison, animals that received FePro labeled CTLs demonstrated hypointense voxels on T2WI and T2*WI around the tumors, as well as in the central part of the tumors. The hypointense regions were more pronounced on T2*WI and were clearly visible as hyperintense areas on the corresponding R2* maps (**Figure 4A** **, lower panel**). Prussian blue staining showed multiple, iron positive cells within both, the periphery and the tumors. Hypointense voxels were present on T2*WI obtained on days 3 and 7. Animals that received labeled control T-cells also demonstrated hypointense voxels on T2*WI and corresponding, increased R2* values (**Figure 4A**, **middle panel**). However, the hypointense regions were not similar to those seen in animals that received magnetically labeled CTLs. **Figure 4B** shows detailed histological analysis of the tumor that received LCTL. Immunohistochemistry confirmed the accumulation of activated human T-cells (CD45RO) at the sites of Prussian blue positive cells (**Figure 5**) in animals that received FePro labeled CTLs. CD45RO positive cells were also seen in tumor that received unlabeled CTLs. (**Supplement materials**, **Figure S5 (S5A and S5B)** also show migration and accumulation of magnetically labeled CTLs to smaller tumor).

### MR Imaging and Histology- Animal Model of Brain Radiation Injury

**Figure 6:**
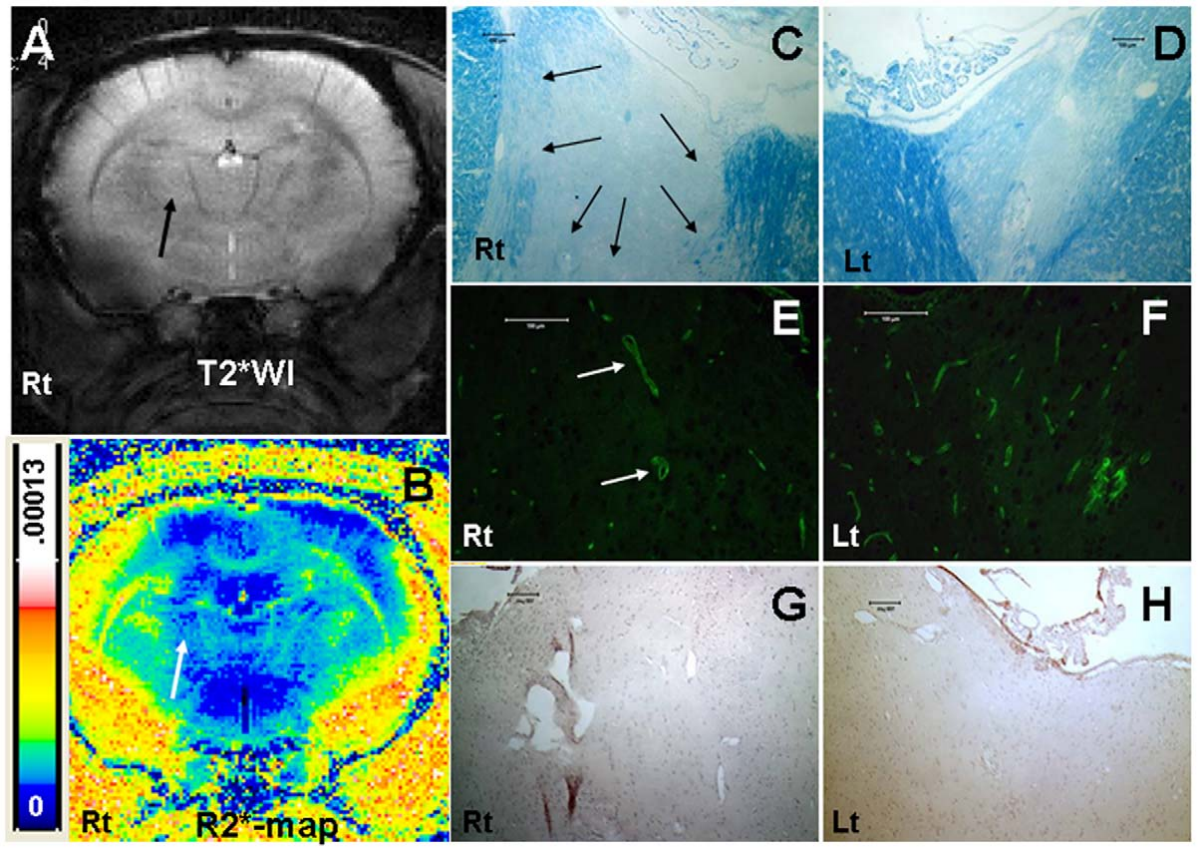
MRI and histology in radiation injury. Representative magnetic resonance imaging and corresponding histochemical analysis of radiation injured (8 weeks after 50Gy focal irradiation) animal that received labeled CTL. (A) T2*-weighted image showed no definite low signal intensity area at the site of radiation injury (arrow) compared to contralateral hemisphere. (B) Corresponding R2* map also showed no high signal intensity area. Luxol fast blue staining revealed extensive loss of myelination at the site of radiation injury (C) compared to that at the corresponding site of contralateral normal hemisphere (D). FITC-tagged tomato lectin staining showed loss of normal blood vessels morphology with evidence of dilatation (arrows) at the corresponding site of radiation injury (demyelinated area) (E), in contrast to the vessel distribution seen in the corresponding site of the contralateral normal hemisphere (F). DAB enhanced Prussian blue staining showed no Prussian blue positive cells at the corresponding radiation injured or contralateral normal hemispheres (G and H). Luxol fast blue, lectin and Prussian blue stained tissues sections were obtained from consecutive slices.

**Figure 7:**
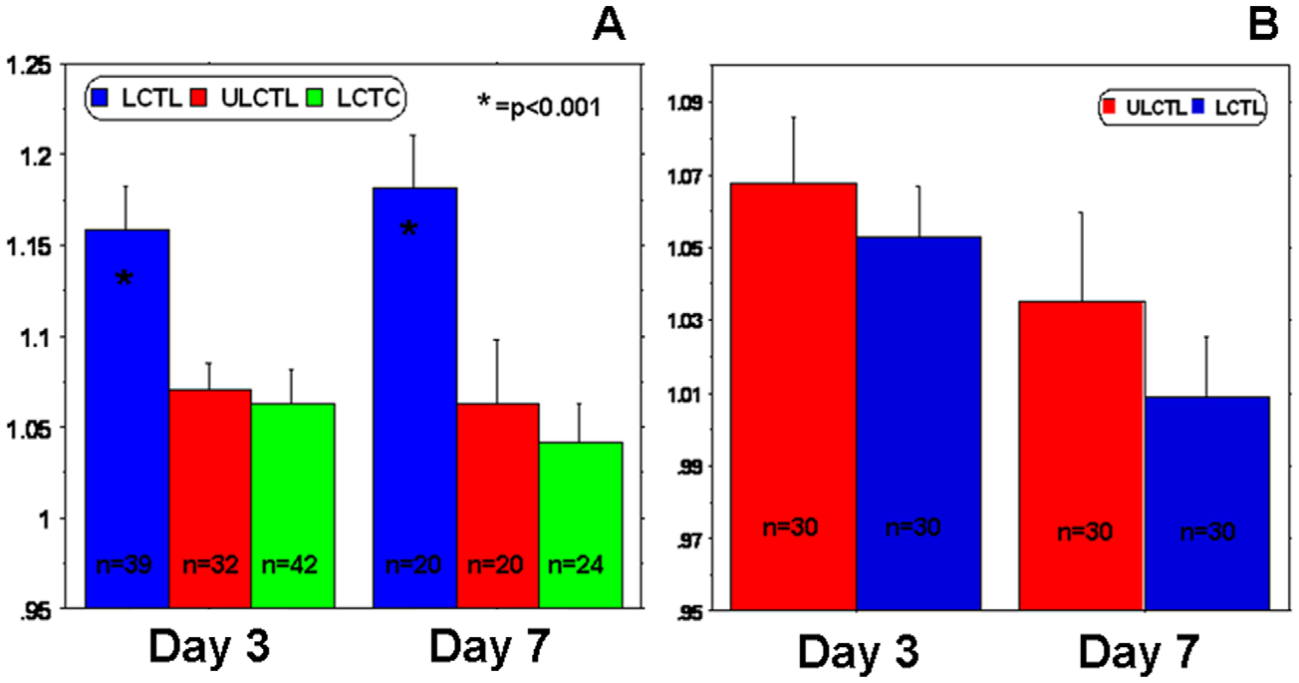
Relaxation parameters in tumors and radiation injuries. (A) Analyses of R2* values normalized to contralateral normal hemisphere (indirect indicator of the accumulation of iron positive cells) showed significantly higher (P = <0.001) accumulation of iron positive cells in tumor that received labeled CTLs. The number of accumulated cells was higher at both, days 3 and 7. n represents the number of sections containing tumors in at least 5 animals. (B) Similar analyses of R2* values normalized to contralateral normal hemisphere showed no difference between the groups of animals that received labeled and unlabeled CTLs. n represents the number of sections included in the analyses from 6 animals (5 sections from each animal, at the level of bregma, 2 sections front and 2 sections behind).

T2WI and T2*WI obtained 8 weeks after irradiation did not show any changes in signal intensity at the sites of radiation injury, as compared to the control, healthy hemispheres. There were no signal intensity changes following intravenous administration of magnetically labeled cells, despite the obvious radiation injury as determined by histological analysis (Luxol fast blue and FITC-labeled tomato lectin staining). Luxol fast blue staining showed extensive loss of myelination at the sites of radiation exposure and lectin staining at the corresponding sites showed dilated blood vessels indicating vascular injury. There were no Prussian blue positive cells detected at the sites of demyelination attributed to radiation injury (**Figure 6**). MR Image analysis demonstrated that, when normalized to the contralateral hemispheres, both R2 and R2* values in tumors of the animals that received labeled CTLs were significantly higher (p = <0.001) compared to that of the animals that received control T-cells and unlabeled CTLs (**Figure 7**). The significant changes were observed at both, day 3 and day 7 after the administration of the cells. On the other hand, there were no significant differences in R2 and R2* values seen in the irradiated hemispheres of the animals that received either labeled or unlabeled CTLs.

## Discussion

The results of this “proof of principle” study demonstrate that incubation of irradiated mature DCs that were primed with U251 tumor cell lysate with T cells can generate fully functional cytotoxic T-cells (CTLs) against human glioma U251 cell line. In addition, produced CTLs can be magnetically labeled and used as cellular MRI probes for delineating sites of the implanted tumors. The results supported our hypothesis that these magnetically labeled CTLs would not accumulate at the sites of radiation injury and could be used to differentiate glioma from radiation injury by cellular MRI. To our knowledge, this is the first study to utilize established methods to produce CTLs *in vitro* that are targeted against a human glioma cell line (U251). In addition, this is the first study to use these sensitized CTLs in conjunction with *in vivo* MRI methods to differentiate implanted tumors from radiation injury in the rat brain. Investigators have used T-cells to target tumors in various experimental and clinical settings [28]–[33]. In another animal model of glioblastoma (U87 cells), investigators have shown the effectiveness of genetically modified T-cells to reduce tumor mass and demonstrated corresponding changes on *in vivo* MRI [31]. Using the same genetically altered T-cells, Yaghoubi et al. have shown the migration and accumulation of CTLs to tumor by PET scanning in a patient with glioblastoma [30]. Rosenberg et al. have shown mild to moderate responses to genetically altered CTL treatment in melanoma patients [32], [34]. In the previous studies, CTLs were collected either from animals (*in vivo* sensitization) or expanded *ex vivo* by genetic manipulation of T-cells to target tumor specific antigens. In our previous study, we used cellular MRI to demonstrate that FePro labeled sensitized splenocytes (containing CTLs and other cells) localized at sites of tumor [17]. The T2WI and T2*WI and the histochemistry demonstrated the presence of Prussian blue positive cells in and around the sites of implanted tumors. In the current study, the accumulation and distribution of magnetically labeled CTLs to the implanted glioma were similar to our previous report [17] and the CTLs were identified not only in the periphery but also within the tumor, as confirmed histologically by the presence of CD45RO positive cells.

Although vaccines against most tumors are under development, dendritic cell-based vaccines have only been used in early clinical trials for glioblastoma multiforme, with varying degrees of success [15], [16]. The basic treatment principle behind these dendritic cell-based vaccines studies was to initiate sensitization of endogenous T-cells by injecting primed dendritic cells, subcutaneously, that would in turn migrate to the nearest draining lymph nodes and sensitize T-cells against the antigen. The sensitized (cytotoxic) T-cells would then target cells that express the antigens, resulting in tumor cell death [35]–[37]. Most of the dendritic cell-based vaccinations were based on priming dendritic cells with the specific tumor antigen. In the current study, we used U251 whole cell lysate, where the specific tumor antigen/s responsible for the sensitization was unknown, to prime the dendritic cells for *in vitro* sensitization of T-cells. However, LDH release studies demonstrated the specificity of the produced CTLs against U251 cells. We also showed the ability to produce CTLs that can function as molecular imaging probes to delineate implanted tumors by MRI. The recently developed rapid one step (i.e., <4 hr) ferumoxides-protamine sulfate method was used to label the CTLs and generate an effective cellular probe compatible with MRI [38].

Animals that received magnetically labeled control T-cells (LCTC) showed smaller numbers of Prussian blue positive cells in the tumors than those that were given LCTLs. However, analysis of R2* values did not show any significant differences from that of the animals that received unlabeled CTLs (ULCTL). On the other hand, the animals that received LCTLs showed large numbers of Prussian blue positive cells and significantly higher R2* values in the tumors. These results indicated that CTLs targeted and accumulated within the tumors as a necessary first step in the development of this approach as a cellular therapy. There was no difference in the distribution of CD45RO positive cells in the tumor in the animals that received LCTL versus ULCTL. These findings indicate that labeling the CTLs with superparamagnetic iron oxides nanoparticles did not alter their ability to home or target the tumors. Moreover, there was no difference in the migration and accumulation specificity between LCTL and ULCTC as shown in our *in vitro* experiments. These results support previous studies demonstrating that magnetic labeling does not alter cell function [27]. The accumulation of magnetically labeled control T-cells to the tumors can be explained in two ways: 1) non-specific accumulation or 2) due to sensitization of the administered labeled control T-cells to tumor antigens (from implanted tumors) during their passage through the spleen. Although the nude rats were used in this study, this kind of migration and priming of the dendritic cells cannot be ruled out.

PET images have high contrast to noise level but are limited by its lower spatial resolution as compared to other tomographic imaging techniques. Genetically altered cytotoxic T-cells have recently been used in the glioblastoma patients where the migration and accumulation of these cells was tracked using PET [30]. However, it is necessary to register the PET to MR images in order to delineate the anatomical sites of the accumulated radiotracer activity. If magnetically labeled cells are used as cellular probes in combination with higher field strength MRI systems with higher spatial resolution, more sophisticated data acquisition approaches and more robust image analysis [39], [40], it would be possible to translate this approach to the bedside for the clinical evaluation of glioblastoma patients. Investigators have already used magnetically labeled cells in a different clinical setting to target the migration of dendritic cells, neural stem cells, bone marrow derived CD34+ cells and islet grafts [41]–[44]. Despite the advancement in imaging modalities, the early detection of residual tumor or metastatic foci in glioblastoma multiforme patients is still controversial, especially in the areas with a mixture of inflammation and malignancy [9], [10], [45], [46]. Since magnetic labeling of cells does not interfere with radionuclide or PET tracer accumulation in genetically engineered cells [47], it will be possible to combine these two approaches to clinically monitor and functionally assess the temporal and spatial migration of cells using the recently introduced PET/MRI systems [48].

### Limitations of the Study

We have used whole tumor cell lysate to prime the dendritic cells, which is a crude and nonspecific approach for sensitizing DC. Investigators have shown effective sensitization of T-cells with profound antitumor effects when CD40 ligand (CD40L) is expressed in tumor cells [49]. Expression of CD40L also helped maturation of dendritic cells. Although expression of CD40 ligand was not analyzed in U251 cells, the presence of this ligand cannot be ruled out. However, our method can be used to prime dendritic cells with any cancer antigen coming from any type of cancer cells. In addition, dendritic cells can be genetically modified to express the specific antigen for sensitization of T-cells. Then, generated sensitized T-cells can be magnetically labeled and used as a probe to target the tumor sites.

Current MR imaging strategies do not allow conclusive determination or differentiation of recurrent or residual glioma from radiation necrosis. Our new proposed method of determining recurrent or residual glioma using magnetically label CTLs may suffer from artifacts due to pre-existing hemorrhage, necrosis and heterogeneity of vascular changes. Of course these artifacts can be circumvented by obtaining an MRI prior to injection of magnetically labeled CTLs and performing image registration followed by subtracting or comparing the pre from post injection images (3 and 7 days following injection) assuming the sites of hemorrhagic and or necrotic foci or pre existing low signal intensity areas will not change dramatically over the time-frame.

## Materials and Methods

### Ethics Statement

All animal experiments were performed according to NIH guideline and the protocol was approved by institutional animal care and user committee (IACUC). The collection of human cord blood, separation of cells and using them in the animal studies were approved by Henry Ford Hospital Institutional Review Board (Protocol # 3287, 4586). The collection process was anonymous (without recording any patient's profile). Written informed consent was obtained to collect the cord blood and the consent process was maintained under IRB-approved security protocol using the IRB-approved consent form.

### Human Glioma U251 Cells

Human glioma cells (U251, a generous gift from Dr. Tom Mikkelsen, Henry Ford Hospital) were grown in Dulbecco's modified eagles medium (DMEM) supplemented with 10% fetal bovine serum (FBS) in 5% CO_2_/95% air at 37°C in a humidified incubator. For implantation in rat brain, cells were harvested and resuspended at a concentration of 8×10^7^ per ml of serum free media; 5 µl of the cell suspension was implanted into the rat brain. Tumor cells were also harvested for generating the whole cell lysates (see below).

### CD14+ and CD2+/CD3+ Cells

Both types of cells were isolated from human cord blood under the approved IRB protocol. CD14+ positive cells were separated from other mononuclear cells (cord blood mononuclear cells were obtained using a Ficoll gradient separation technique) by magnet activated cell sorter (MACS) using magnetic beads conjugated with anti-CD14 antibodies. CD14 depleted cells were further incubated with anti-CD2 antibodies conjugated to magnetic beads to separate CD2+/CD3+ cells. Collected CD14+ cells were further differentiated into dendritic cells. However, based on the need of the experiments CD2+/CD3+ cells were either cryopreserved or used immediately. For sensitization of collected T-cells using mature primed DCs, cells collected from same donor were used. There was no variability observed from donor to donor with respect to mature DCs production and sensitization of T-cells.

### Preparation of Tumor Cell Lysate

U251 human glioma cells were harvested and washed twice in ice cold sterile H_2_O. The cell pellets (1×10^8^) were repeatedly frozen in liquid nitrogen and thawed at 37°C to disintegrate the cells. Cell lysates were resuspended in 5 ml of sterile H_2_O, centrifuged at 4°C for 15 min using high speed, and the supernatant was collected and passed through a 0.45 µm filter. Total amount of protein in the collected fluid was determined by standard colorimetric methods using a commercially available protein concentration assay kit (Pierce, Rockford, IL).

### Preparation of Tumor Cell Lysate-Pulsed Mature Dendritic Cells

CD14+ cells were resuspended at the concentration of 3−5×10^5^ cell/ml in RPMI 1640 media containing 10% FBS, 25 ng/ml of IL-4, 50 ng/ml of G-CSF (granulocyte colony-stimulating factor) and incubated in 5% CO_2_/95% air at 37°C in a humidified incubator for 4 days to make immature dendritic cells. During this incubation period 1/2 of the media was replaced with 2/3^rd^ of fresh media containing cytokines on day 3. On day 5, suspended and loosely adherent cells were collected, centrifuged and resuspended in fresh RPMI 1640 media containing 10% FBS, 25 ng/ml of IL-4, 50 ng/ml of GM-CSF and 50 µg/ml of tumor cell lysate at 5×10^5^ cells/ml. The cells were thoroughly mixed and further incubated for 4 days. At the end of the 4 day priming, cells were collected and resuspended in fresh media containing 10% FBS, 25 ng/ml of IL-4, 50 ng/ml of GM-CSF and 100 ng/ml of TNF-α and incubated for additional 4 days. Total incubation time from the collection of CD14+ cell to generating primed mature dentritic cells was 12 days. Expression markers specific for dendritic cells (such as CD14, CD86, CD83 and HLA-DR) were assessed by flow cytometry before and after addition of TNF- α.

### Sensitization of Isolated T-Cells

Either fresh or cryopreserved T-cells tha were isolated from cord blood were cultured overnight in RPMI 1640 media containing 10% FBS, sodium pyruvate, non-essential amino acids, L-glutamine and 10 ng/ml of IL-2 and then co-cultured with irradiated (35Gy) cell lysate-pulsed mature dendritic cells for 6–7 days. The initial ratio of T-cell to dendritic cell was 10:1. T-cell proliferation was monitored every day and based on the cell density, fresh media was added to the co-culture. T-cell proliferation was determined by a proliferative activity assay (**MTT,** (3-(4,5-Dimethylthiazol-2-yl)-2,5-diphenyltetrazolium bromide, a tetrazole) assay) on days 1, 3 and 5 and the values were normalized using the corresponding control cells. After incubation for 6 days the T-cells were collected, washed and resuspended in serum free media at a concentration of 4×10^6^ cells/ml and labeled with iron oxides. Phenotypical expression of different T-cell markers (CD3, CD4, CD8, and CD25) was determined by a flow cytometer (LSR II, BD Bioscience) before and after sensitization, as well as after the magnetic labeling of CTLs. Viability and secondary proliferation (MTT) capacity of magnetically labeled and unlabeled CTLs were also determined.

### Preparation of Ferumoxides-Protamine Sulfate (FePro) Complex and Labeling of Cells

Commercially available, FDA-approved super paramagnetic iron oxides (SPIO) ferumoxides suspension (Feridex IV ®, Bayer-Schering Pharmaceuticals Inc, Wayne, New Jersey) contains particles that are approximately 80–150 nm in size and has a total iron content of 11.2 mg/ml (11.2 µg/µl of iron). Protamine sulfate (American Pharmaceuticals Partner Inc. Schaumburg, IL), supplied at 10 mg/ml, was prepared as a fresh stock solution of 1 mg/ml in distilled water at the time of use. We have labeled CTLs using a modified published method (also described in supplement), where instead of making FePro complexes prior to adding to the cell suspension, we now add ferumoxides (100 µg/ml) directly to the cell suspension in serum free media and then add protamine sulfate (3 µg/ml) [38]. The FePro complexes are formed in the cell suspension. After 15 minutes of incubation in serum free media, an equal volume of complete media (containing serum) was added to the cell suspension and further incubated for 4 hours. The cells were then collected, washed with sterile PBS and resuspended at the specific concentration for injection.

### Cellular Viability, Phenotypic Analysis, Labeling Efficiency and Mean Iron Concentration of FePro Labeled Cells

After labeling, the cells were washed two times with sterile phosphate buffered saline (PBS) and resuspended in PBS at a concentration of 3×10^7^ cells/ml. Cell viability was determined by trypan blue exclusion assay. Expression markers specific for CTLs were analyzed by flow cytometer in labeled and unlabeled cells, using fluorescently conjugated antibodies. Labeling efficiency was determined by Prussian blue staining and intracellular iron content that was determined by our published method [50]. Iron concentration was expressed as average picogram of iron/cell.

### Determination of Specificity of Labeled and Unlabeled CTLs

To determine whether produced CTLs have the specificity to target U251 cells *in vitro* and to determine whether FePro labeling alters this specificity, 100k of U251 cells were plated in 24 well plates and cultured until reaching 90% confluence. Then, either 100k or 200k of control T-cells, unlabeled CTLs or labeled CLTs were added separately to the wells containing U251 cells. The interaction (accumulation of the added T-cells around U251 cells) of the added cells with U-251 cells was photomicrographed at 0 and 18 hours of co-culture. Both unlabeled and labeled CTLs were also subjected to secondary stimulation by incubating with irradiated primed dendritic cells and cell proliferation was determined as described above.

To determine the cytotoxic specificity of the produced CTLs to U251 cells, 200k CTLs (sensitized to U251 cell lysate) were co-cultured overnight with U251 (100k) or human breast cancer cell (MBA-MD-231, 100k) and the released lactate dehydrogenase (LDH) was determined by a commercially available membrane integrity assay kit (Cyto Tox-ONE, Promega corp, WI). LDH levels indicate cytotoxicity since LDH is released to the media once cell membranes are damaged.

To determine *in vivo* specificity of produced CTLs, magnetically labeled CTLs sensitized to U251 cells were intravenously injected in mice bearing either subcutaneous MBA-MD-231 breast cancer (n = 2) or U251 glioma (n-2). Three days following administration of labeled CTLs all animals underwent *in vivo* MRI. On day 7 after administration of cells animals were euthanized, perfused and tumors along with surrounding tissues were collected for histochemical analysis (Prussian blue staining).

### U251 Glioma Rat Model

Following NIH guidelines under the institutional animal care and user committee (IACUC) approved protocol, 48 nude rats were inoculated intra-cerebrally as follows: *Implantation*: Animals were anesthetized with 100 mg/kg of ketamine and 10 mg/kg of xylazine i.p., the surgical zone was swabbed with betadine solution, eyes coated with Lacri-lube and the animal was immobilized in a small animal stereotactic device (Kopf, Cayunga, CA). After draping, a 1-cm incision was made 2 mm to the right of the midline and 1 mm retro-orbitally. The skull was exposed with cotton-tip applicators and an HP-4 dental drill bit was used with a micromanipulator to drill a hole 3 mm to the right of the bregma, taking care not to penetrate the dura. A #2701 10 µL Hamilton syringe with a #4 point, 26 gauge-needle containing tumor cells (4×10^5^) was initially lowered to a depth of 3.5 mm, then raised back up to a depth of 2.5 mm. The U251 cells were injected stepwise at a rate of 0.5 µL/30 sec until the entire volume was injected. During and after the injection, careful note was made of any reflux from the injection site. After completing the injection, the needle was withdrawn in a stepwise manner and the surgical hole sealed with bone wax. Finally, the skull was swabbed with betadine before suturing the skin over the injection site. Animals were intravenously administered unlabeled CTLs, magnetically labeled control T-cells or magnetically labeled CTLs on day 14 post-tumor implantation and MR images were obtained on days 3 and 7 after the administration of cells. There were two time points (day 3 and 7 after injection) in each group (labeled control T-cells, unlabeled CTLs and labeled CTLs). Results from a few rats were discarded due to bleeding and failure to develop tumors, however at least 5 animals were included at each time point for each group.

### Radiation Injury to the Rat Brain

Animals (24 rats) were anesthetized using 100 mg/kg of ketamine and 10 mg/kg of xylazine i.p. and restrained in a stereotactic head holder. The site of irradiation was chosen to be at 3 mm right from bregma. Rats were irradiated sterotactically using a single collimated dorso-ventral beam of 6MV X-rays with 1.5 mm thick bolus material placement so that 95–100% of the central axis dose was delivered to the target volume [51]. The volume of brain irradiated was a cylinder of radius 3 mm at the 80% isodose boundary. The dose rate of 6 MV X-rays is 6 Gy/min using a 75-cm source-to-skin surface distance. The radiation injury was induced using 50Gy [52]. Based on our preliminary data and the previous experiences (with radiation injuries which are obvious in 8 weeks), the animals were intravenously injected with either unlabeled or labeled CTLs on day 56 after radiation exposure and MR images were obtained on days 59 and 63. There were 6 animals studied at each time (59 and 63 days) point for each of 4 groups.

### *In Vivo* MR Imaging Studies

An appropriate state of anesthesia was obtained with isoflurane (3% for induction, 0.7% to 1.5% for maintenance in a 2:1 mixture of N_2_:O_2_). The anesthetized rats were placed in a 7 Tesla, 20 cm bore superconducting magnet (Magnex Scientific, Abingdon, England) interfaced to a Bruker Avance console (Bellerica, MA). A 12 cm self-shielded gradient set with maximum gradients of 25 gauss/cm was used. The radio frequency (rf) pulses were applied by a 7.5 cm diameter saddle transmit coil actively decoupled from the 3.2 cm diameter surface receive coil which was positioned over the center line of the animal skull. Stereotaxic ear bars were used to minimize movement during the imaging procedure. Rectal temperature was maintained at 37±0.5°C using a feedback controlled water bath. After positioning using a triplanar FLASH sequence, MR studies were performed using T2WI and T2*WI. For detection of iron oxide labeled cells, scans typically employ gradient-echo imaging methods to enhance T2*-weighting effects. The following parameters were used to acquire MRI: T2 weighted images (T2WI) were obtained using a standard two-dimensional Fourier transformation (2DFT) multi-slice (13–15 slices) multi-echo (6 echoes) MRI sequences with TEs of 15, 30, 45, 60, 75 and 90 msec and a TR of 3000 msec using a 32 mm FOV, 1 mm slice thickness, 256×256 matrix, and NEX  = 2. The T2* weighted images (T2*WI) were obtained using a standard multislice (13–15 slices) multi gradient-echo (4 echoes) MRI using TEs of 5, 10, 15, and 20 msec and a TR of 3000 msec. The images were acquired using a 32 mm FOV, 1 mm slice thickness, 256×256 matrix, and NEX  = 2. We understand that post contrast T1WI will clearly delineate the tumor margins as compared to T2WI. However, post contrast T1WI relies on the BBB disruption or permeable tumor vasculatures,that may not be present in the small number of cells that migrate away from the typical tumor margin. These microscopic satellite islands of tumors can not be delineated with post-contrast T1WI because these tumor cells have co-opted vessels and have not resulted in BBB disruption. On the other hand, encircling all the high signal area on T2WI will include both the tumor as well as surrounding edematous area that may contain invasive tumor cells as well as the migrated CTLs.

### Analysis of MRI Images

Transverse relaxation rate R2 (1/T2) and R2* (1/T2*) maps were constructed from their respective multi-echo T2 and T2* image sets. The T2 and T2* decay was estimated from a least square fit on a pixel-by-pixel basis using an exponential model of the time series extracted from the multi-echo T2-weighted spin-echo and gradient-echo images, respectively. The Eigentool image analysis software package, developed at the Radiology Image Analysis Laboratory of the Henry Ford Hospital, Detroit, MI (http://www.radiologyresearch.org/eigentool.htm) that includes an implementation of this estimation algorithm was used. The resultant values were inverted to generate the R2 and R2* maps. The R2 and R2* values in the tumors or radiation injury and contralateral brains were determined by hand drawn ROIs. The R2 and R2* values were calculated from every section that contained tumor. For radiation injury the ROIs were drawn for a total of 5 sections of the brain (2 sections anterior to bregma, two sections posterior to bregma and one at the site of bregma). The R2 and R2* values in the tumors or radiation injury areas were normalized to the corresponding contralateral brain by creating the ratios of the tumor and radiation injury to the normal brain (Tumor/Contralateral brain, Radiation injury/Contralateral brain). The R2 and R2* values were determined by an operator who was masked to the animal groups.

### Histology and Immunohistochemistry

Animals were euthanized immediately after the last MR imaging session to dissect the tissues via 150–200 mg/kg of pentobarbital (administered by intravenous or intra-peritoneal injection) and then perfused with 100 mL of saline and 100 ml of 3% paraformaldehyde for histological analysis. The whole brain was collected and fixed in 4% paraformaldehyde and 3% sucrose. The fixed brain was placed in a 200–400 g coronal rat-brain matrix (Activational Systems Inc., Warren, MI) and cut into 1-mm blocks. Blocks grossly containing and adjacent the tumor or radiation injury were processed and paraffin embedded. Some of the tissues were also processed as frozen sections. The embedded blocks were cut into serial 10 µm sections for immunohistochemical and Prussian blue staining. Consecutive tissue sections were evaluated by the standard immunohistochemical techniques for the presence of activated T-cells. Sections of the brain from both the tumor and radiation injury areas were stained with Prussian blue to determine the number of migrated iron positive cells at the site of lesions.

### Data Analysis

All data are expressed as mean ± standard deviation (unless indicated differently). Comparisons of measurements among the tumor implanted groups and the radiation injury groups were performed using repeated measurement analysis of variance (ANOVA) followed by Fisher's PLSD post hoc test. The p-values equal to or less than 0.05 were considered significant.

## Supporting Information

Table S1 Cell surface markers expression in CD14+ cells during differentiation to mature dendritic cells in the presence of G-CSF and IL-4 at different concentrations. In an attempt to optimize the conditions for maturation of dendritic cells, after collection of CD14+ cells by the magnetic cell sorting MACS® technique, cells were incubated for 8 days in dendritic cell media containing various relative and absolute amounts of G-CSF (granulocyte colony stimulating factor) and IL-4 (interleukin 4), as shown in Table 1. At the end of day 8 in culture, TNF-α at 100ng/ml was added to the cells and incubated further for 3 days. Phenotypical expression of different markers was determined at different time points. There was no significant difference in the expression of different markers among the different composition of cytokines. We opted to use 50ng/ml of G-CSF, 25ng/ml of IL-4 and 100ng/ml of TNF-α for all the subsequent experiments involving primed mature dendritic cells. The data are expressed as mean ± standard deviation from sample sizes of 2 to 4. We have assessed HLA-DR positive cells to determine monocyte-derived DCs.
(0.04 MB DOC)

Figure S1 MTT assay. MTT assay shows significantly (p = <0.01) higher T-cell growth in the presence of primed irradiated dendritic cells (PIDC).
(1.77 MB TIF)

Figure S2 Labeling of CTLs. Commercially available, FDA-approved SPIO, ferumoxides suspension, (Feridex IV ®, Bayer-Schering Pharmaceuticals Inc, Wayne, New Jersey) contains particles approximately 80–150 nm in size and has a total iron content of 11.2 mg/ml (11.2 of iron µg/µl). Protamine sulfate (American Pharmaceuticals Partner Inc. Schaumburg, IL), supplied at 10 mg/ml, was prepared as a fresh stock solution of 1 mg/ml in distilled water at the time of use. CTLs were collected in tubes, washed two times with serum free media to get rid of the serum and resuspended at the concentration of 4×106 per ml of serum free RPMI-1640 media containing L-glutamine, sodium pyruvate and essential amino acids. Then 100 µg (9 µl of solution from the bottle) of ferumoxides for each ml of cell suspension were added to the tubes and mixed well. Three µl (3 µg of protamine sulfate) of freshly prepared protamine sulfate was then added to the tube containing cell suspension and ferumoxides, and mixed well. The mixtures were transferred to 6-well plates at a concentration of 10×106 cell per well, i.e. 2.5 ml per well and allowed to react for 15 minutes at 37°C in a tissue culture incubator. After 15 minutes, equal volume of complete T-cell media (RPMI1640, 10% FBS, 10 ng/ml IL-2, L-glutamine, sodium pyruvate and essential amino acid) was added to the wells and incubated for 4 hours. After incubation, cells were collected, washed and cytospin slides were made. Prussian blue staining was performed to determine the labeling efficiency. DAB enhanced Prussian blue staining of unlabeled (A) and ferumoxides-protamine sulfate labeled (B) CTLs. Note the extensive labeling of CTLs by our newer procedure.
(4.55 MB TIF)

Figure S3 Production and specificity of CTLs against U87 and 9L glioma. To determine whether the *ex vivo* method for priming DC and sensitization of T-cells could be used to create CTLs against U87 (human) and 9L (rat) glioma cell lines. U87 and 9L tumor cell lysate primed mature DCs were produced using cord blood derived CD14+ cells. These cells were then irradiated at 35Gy and co-cultured with cord blood derived CD2+/CD3+ cells (T-cells) according to our described method. After 6 days of sensitization, respective CTLs (200k) were added to wells containing 9L and U87 cells. Same numbers of control T-cells (200k, non-sensitized) were also added to the wells containing 9L or U87 cells. Interaction (accumulation of added T-cells around the U87 or 9L cells) of the added cells was photomicrographed at 18 hours. Left column: Morphology of 9L (upper panel) and U87 (lower panel) after incubation with 200k of CTCs (control T-cells) at 18 hours. There is no change in the morphology of 9L and U87 cells, and CTCs (small round cells) appear to be passively “sitting”on the tumor cells even after 18 hours of incubation. Right column: Morphology of 9L (upper row) and U87 (lower row) after incubation with 200k of respective CTLs (sensitized T-cells) at 18 hours. Specific accumulations of CTLs around the tumor cells are seen after 18 hours for both cell types. Compared to the 9L and U87 cells cultured with CTCs (left column), there are dramatic changes in the morphology of 9L and U87 cells incubated with CTLs.
(3.45 MB TIF)

Figure S4 *In vivo* specificity of CTLs. To determine *in vivo* specificity of produced CTLs, magnetically labeled CTLs sensitized to U251 cells were intravenously injected in mice bearing either subcutaneous MBA-MD-231 breast cancer (n = 2) or U251 glioma (n-2) tumors. Three days following the administration of labeled CTLs all animals underwent *in vivo* MRI. Seven days after the administration of cells animals were euthanized, perfused and tumors along with the surrounding tissues were collected for histochemical analysis (Prussian blue staining). Increased number of magnetically labeled CTLs were observed in subcutaneous gliomas as compared to that of breast cancers. The labeled CTLs accumulated not only at the periphery, but also in the deeper parts of glioma tumors. Similar accumulation was not observed in breast cancer tumors, which indicates *in vivo* specificity of generated CTLs. Figure S4A: Representative case of U251 tumor. Gradient echo (GRE) MRI with two different echo times (TE), 10 ms (A) and 20 ms (B), shows low signal intensity areas along the peripheral parts of the subcutaneously implanted U-251 glioma (Arrows on A and B). DAB enhanced Prussian blue staining shows numerous iron positive cells (dark brown), not only in the peripheral parts (C, D, arrows), but also in the deeper parts of the tumor (C, E, arrows). (C) Two separate photomicrographs were combined together to show the extent of accumulated iron positive cells (magnification 10x). D and E represent magnified images (magnification 25x) of the boxed areas. Figure S4B: Representative case of MBA-MD-231 tumor. Gradient echo (GRE) MRI with two different echo times (TE), 10 ms (A) and 20 ms (B), shows no definite low signal intensity areas along the peripheral parts of the subcutaneously implanted MBA-MD-231 breast cancer tumor. However, large low signal intensity areas were seen within the tumors (Arrows on A and B), which are thought to be due to hemorrhage. DAB enhanced Prussian blue staining shows no definite iron positive cells at the peripheral parts (C, D) of the tumor, however, there are multiple areas of hemorrhagic foci (RBC) seen in the deeper parts of the tumor (E, arrows). A few iron positive cells, that could possibly be injected CTLs, are seen in the deeper part of the tumor (E, arrow head). (C) Magnification 10x. (D and E) Magnification 25x.
(7.41 MB TIF)

Figure S5 Detection of smaller U251 tumor. Magnetically labeled CTLs were injected intravenously in rats bearing U251 implanted tumor (n = 3) on day 7 after the implantation. Both T2-weighted (T2WI) and T2*-weighted (T2*WI) images were acquired on day 3 and 7 after IV administration of CTLs. Following the last MRI, animals were euthanized and their brains including tumors were collected after perfusion and prepared for histochemical analysis. Images from representative experiment demonstrate migration and accumulation of administered CTLs in the small tumor. Figure S5A: T2WI shows high signal intensity areas (arrows) at the site that are considered to be the growing tumor at day 10 (A) and day 14 (B) following the implantation of tumor (day 3 and 7 after IV administration of labeled CTLs). Note the extension of tumor along and below the corpus callosum. T2*WI shows low signal intensity at the sites thought to be growing tumor (arrows) at day 3 (C) and day 7 (D) after the administration of labeled CTLs. Note the low signal areas that are indeed within the high signal areas seen on T2WI. The low signal area seen in the cortex (thick arrow) might be due to needle track and hemorrhage during implantation of tumor. Figure S5B: DAB enhanced Prussian blue (A, B) and CD45RO (C) staining from consecutive sections showing the accumulated iron positive cells (dark brown) in the small tumor mass (A, 10x) and (B, 40X). CD45RO staining shows multiple activated T-cells within the tumor (arrows, Magnification 40X).
(9.53 MB TIF)
