# Supplementary material for: Differentiation of Glioma and Radiation Injury in Rats Using In Vitro Produce Magnetically Labeled Cytotoxic T-Cells and MRI
Source: PLoS One. 2010 Feb 26;5(2):e9365. doi: 10.1371/journal.pone.0009365 (PMC2829084; doi:10.1371/journal.pone.0009365)
Supplement: Table S1 — Cell surface markers expression in CD14+ cells during differentiation to mature dendritic cells in the presence of G-CSF and IL-4 at different concentrations. In an attempt to optimize the conditions for maturation of dendritic cells, after collection of CD14+ cells by the magnetic cell sorting MACS® technique, cells were incubated for 8 days in dendritic cell media containing various relative and absolute amounts of G-CSF (granulocyte colony stimulating factor) and IL-4 (interleukin 4), as shown in Table 1. At the end of day 8 in culture, TNF-α at 100ng/ml was added to the cells and incubated further for 3 days. Phenotypical expression of different markers was determined at different time points. There was no significant difference in the expression of different markers among the different composition of cytokines. We opted to use 50ng/ml of G-CSF, 25ng/ml of IL-4 and 100ng/ml of TNF-α for all the subsequent experiments involving primed mature dendritic cells. The data are expressed as mean ± standard deviation from sample sizes of 2 to 4. We have assessed HLA-DR positive cells to determine monocyte-derived DCs. (0.04 MB DOC) [file pone.0009365.s001.doc]

**Table S1:** cell surface markers expression in CD14+ cells during differentiation to mature dendritic cells in the presence of G-CSF and IL-4 at different concentrations.

| **Cytokines (ng/ml)** | **Day 4 in media** | | | | **Day 8 in media** | | | | **Day 3 after adding TNF-α** | | | |
| --- | --- | --- | --- | --- | --- | --- | --- | --- | --- | --- | --- | --- |
| **G-CSF/IL-4** | **CD14** | **CD86** | **CD83** | **HLA-DR** | **CD14** | **CD86** | **CD83** | **HLA-DR** | **CD14** | **CD86** | **CD83** | **HLA-DR** |
| **25/25** | .11±.02 | 81.54±3.80 | 2.72±1.66 |  | 1.91±2.17 | 84.78±4.86 | .75±.12 |  | .02±.02 | 94.44±3.93 | 59.02±8.00 |  |
| **50/25** | .06±.00 | 77.95±3.59 | 1.80±1.23 | 82.37±0.85 | 1.10±1.23 | 91.34±2.55 | .45±.09 | 87.24±1.10 | .04±.01 | 93.44±5.58 | 55.80±3.80 | 95.95±2.76 |
| **50/50** | .15±.06 | 80.91±2.97 | 2.18±1.54 |  | 2.41±2.65 | 89.79±2.77 | .43±.09 |  | .07±.06 | 93.59±5.11 | 56.47±6.53 |  |
| **100/50** | .05±.01 | 77.99±3.50 | 2.00±1.50 |  | 1.84±2.07 | 87.76±2.13 | .38±.08 |  | .04±.02 | 94.99±3.34 | 65.08±10.53 |  |
| **100/100** | .14±.05 | 81.90±2.59 | 2.10±1.25 |  | 1.91±2.17 | 88.24±2.35 | .35±.04 |  | .01±.01 | 94.61±3.93 | 54.52±6.60 |  |
