# Supplementary figures and images for: Differentiation of Glioma and Radiation Injury in Rats Using In Vitro Produce Magnetically Labeled Cytotoxic T-Cells and MRI
Source: PLoS One. 2010 Feb 26;5(2):e9365. doi: 10.1371/journal.pone.0009365 (PMC2829084; doi:10.1371/journal.pone.0009365)

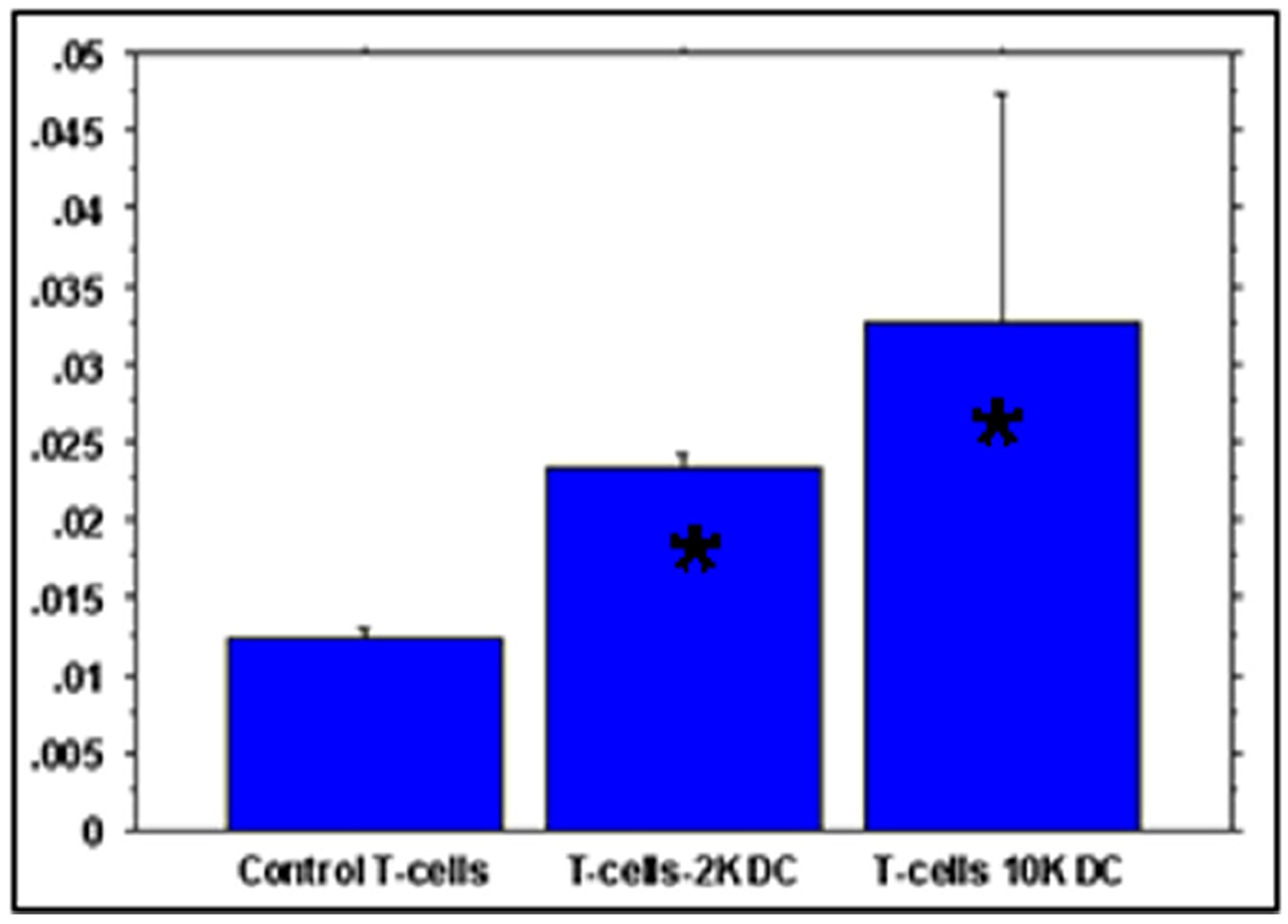

Supplement: Figure S1 — MTT assay. MTT assay shows significantly (p = <0.01) higher T-cell growth in the presence of primed irradiated dendritic cells (PIDC). (1.77 MB TIF) [file pone.0009365.s002.tif]

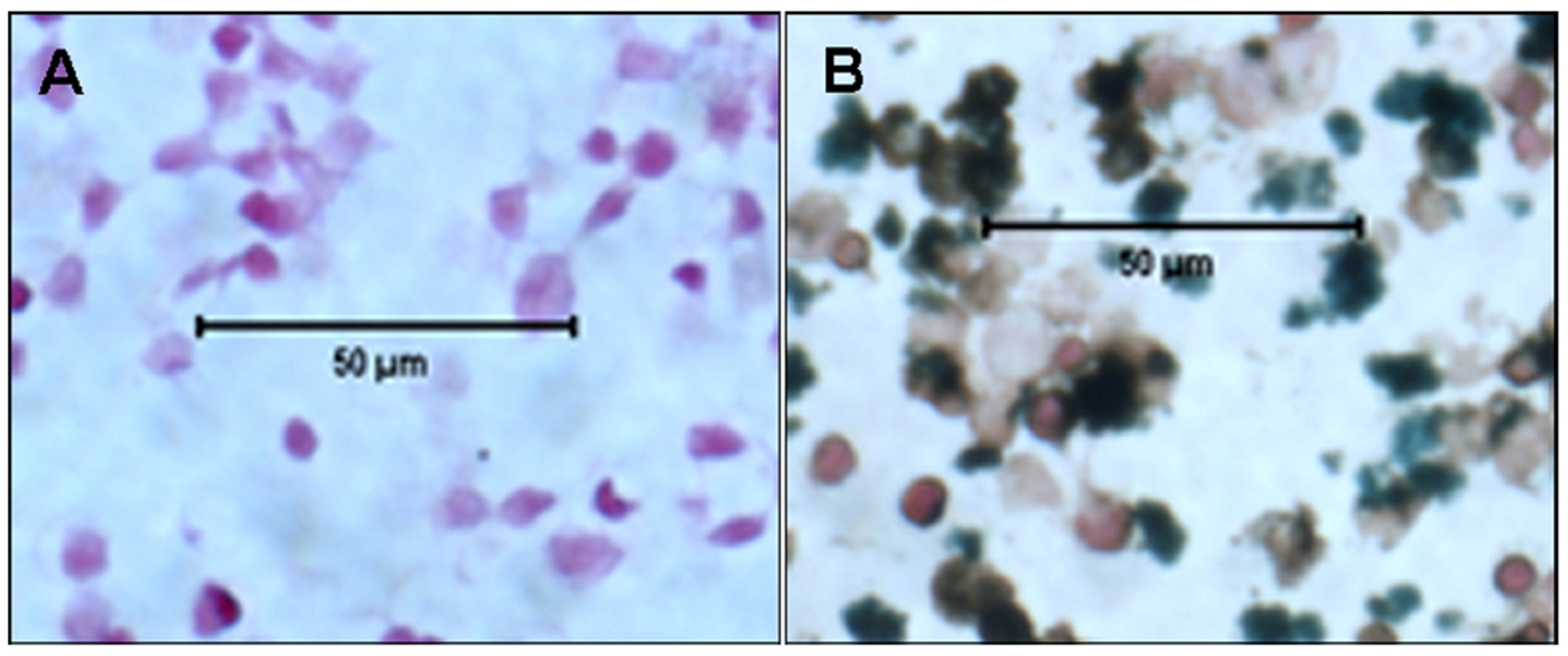

Supplement: Figure S2 — Labeling of CTLs. Commercially available, FDA-approved SPIO, ferumoxides suspension, (Feridex IV ®, Bayer-Schering Pharmaceuticals Inc, Wayne, New Jersey) contains particles approximately 80–150 nm in size and has a total iron content of 11.2 mg/ml (11.2 of iron µg/µl). Protamine sulfate (American Pharmaceuticals Partner Inc. Schaumburg, IL), supplied at 10 mg/ml, was prepared as a fresh stock solution of 1 mg/ml in distilled water at the time of use. CTLs were collected in tubes, washed two times with serum free media to get rid of the serum and resuspended at the concentration of 4×10^6^ per ml of serum free RPMI-1640 media containing L-glutamine, sodium pyruvate and essential amino acids. Then 100 µg (9 µl of solution from the bottle) of ferumoxides for each ml of cell suspension were added to the tubes and mixed well. Three µl (3 µg of protamine sulfate) of freshly prepared protamine sulfate was then added to the tube containing cell suspension and ferumoxides, and mixed well. The mixtures were transferred to 6-well plates at a concentration of 10×106 cell per well, i.e. 2.5 ml per well and allowed to react for 15 minutes at 37°C in a tissue culture incubator. After 15 minutes, equal volume of complete T-cell media (RPMI1640, 10% FBS, 10 ng/ml IL-2, L-glutamine, sodium pyruvate and essential amino acid) was added to the wells and incubated for 4 hours. After incubation, cells were collected, washed and cytospin slides were made. Prussian blue staining was performed to determine the labeling efficiency. DAB enhanced Prussian blue staining of unlabeled (A) and ferumoxides-protamine sulfate labeled (B) CTLs. Note the extensive labeling of CTLs by our newer procedure. (4.55 MB TIF) [file pone.0009365.s003.tif]

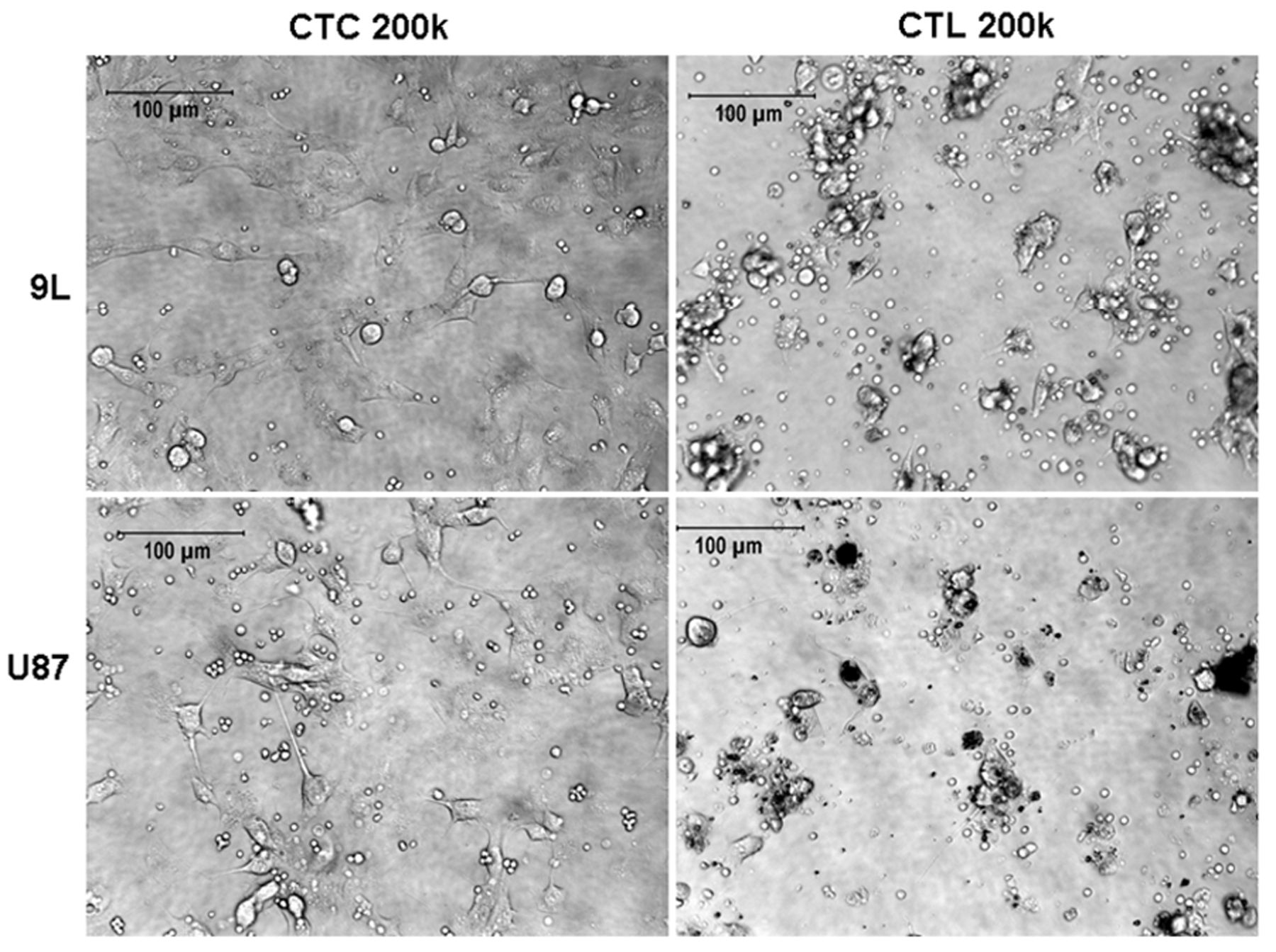

Supplement: Figure S3 — Production and specificity of CTLs against U87 and 9L glioma. To determine whether the ex vivo method for priming DC and sensitization of T-cells could be used to create CTLs against U87 (human) and 9L (rat) glioma cell lines. U87 and 9L tumor cell lysate primed mature DCs were produced using cord blood derived CD14+ cells. These cells were then irradiated at 35Gy and co-cultured with cord blood derived CD2+/CD3+ cells (T-cells) according to our described method. After 6 days of sensitization, respective CTLs (200k) were added to wells containing 9L and U87 cells. Same numbers of control T-cells (200k, non-sensitized) were also added to the wells containing 9L or U87 cells. Interaction (accumulation of added T-cells around the U87 or 9L cells) of the added cells was photomicrographed at 18 hours. Left column: Morphology of 9L (upper panel) and U87 (lower panel) after incubation with 200k of CTCs (control T-cells) at 18 hours. There is no change in the morphology of 9L and U87 cells, and CTCs (small round cells) appear to be passively “sitting”on the tumor cells even after 18 hours of incubation. Right column: Morphology of 9L (upper row) and U87 (lower row) after incubation with 200k of respective CTLs (sensitized T-cells) at 18 hours. Specific accumulations of CTLs around the tumor cells are seen after 18 hours for both cell types. Compared to the 9L and U87 cells cultured with CTCs (left column), there are dramatic changes in the morphology of 9L and U87 cells incubated with CTLs. (3.45 MB TIF) [file pone.0009365.s004.tif]

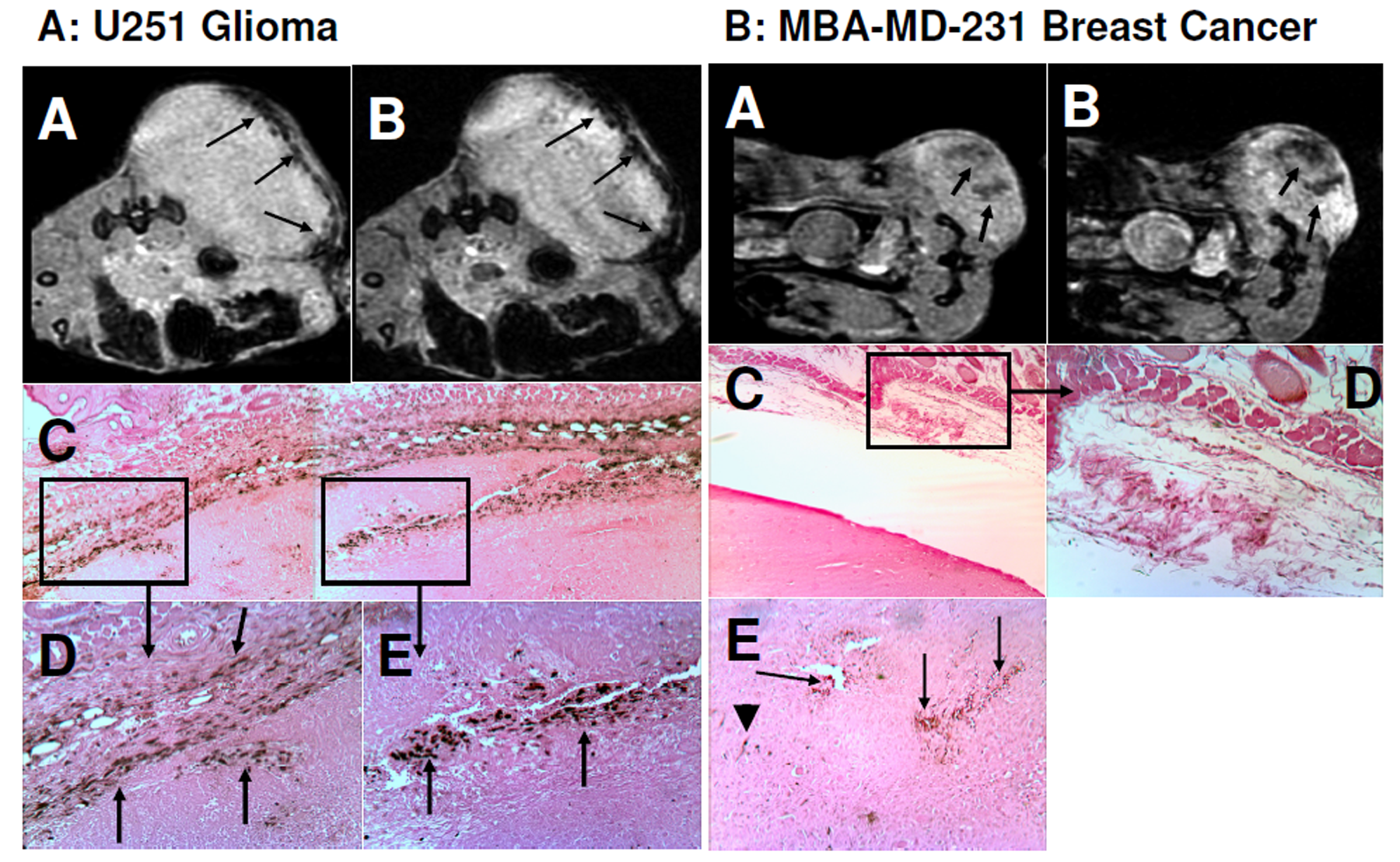

Supplement: Figure S4 — In vivo specificity of CTLs. To determine in vivo specificity of produced CTLs, magnetically labeled CTLs sensitized to U251 cells were intravenously injected in mice bearing either subcutaneous MBA-MD-231 breast cancer (n = 2) or U251 glioma (n-2) tumors. Three days following the administration of labeled CTLs all animals underwent in vivo MRI. Seven days after the administration of cells animals were euthanized, perfused and tumors along with the surrounding tissues were collected for histochemical analysis (Prussian blue staining). Increased number of magnetically labeled CTLs were observed in subcutaneous gliomas as compared to that of breast cancers. The labeled CTLs accumulated not only at the periphery, but also in the deeper parts of glioma tumors. Similar accumulation was not observed in breast cancer tumors, which indicates in vivo specificity of generated CTLs. Figure S4A: Representative case of U251 tumor. Gradient echo (GRE) MRI with two different echo times (TE), 10 ms (A) and 20 ms (B), shows low signal intensity areas along the peripheral parts of the subcutaneously implanted U-251 glioma (Arrows on A and B). DAB enhanced Prussian blue staining shows numerous iron positive cells (dark brown), not only in the peripheral parts (C, D, arrows), but also in the deeper parts of the tumor (C, E, arrows). (C) Two separate photomicrographs were combined together to show the extent of accumulated iron positive cells (magnification 10x). D and E represent magnified images (magnification 25x) of the boxed areas. Figure S4B: Representative case of MBA-MD-231 tumor. Gradient echo (GRE) MRI with two different echo times (TE), 10 ms (A) and 20 ms (B), shows no definite low signal intensity areas along the peripheral parts of the subcutaneously implanted MBA-MD-231 breast cancer tumor. However, large low signal intensity areas were seen within the tumors (Arrows on A and B), which are thought to be due to hemorrhage. DAB enhanced Prussian blue staining shows no defin [file pone.0009365.s005.tif]

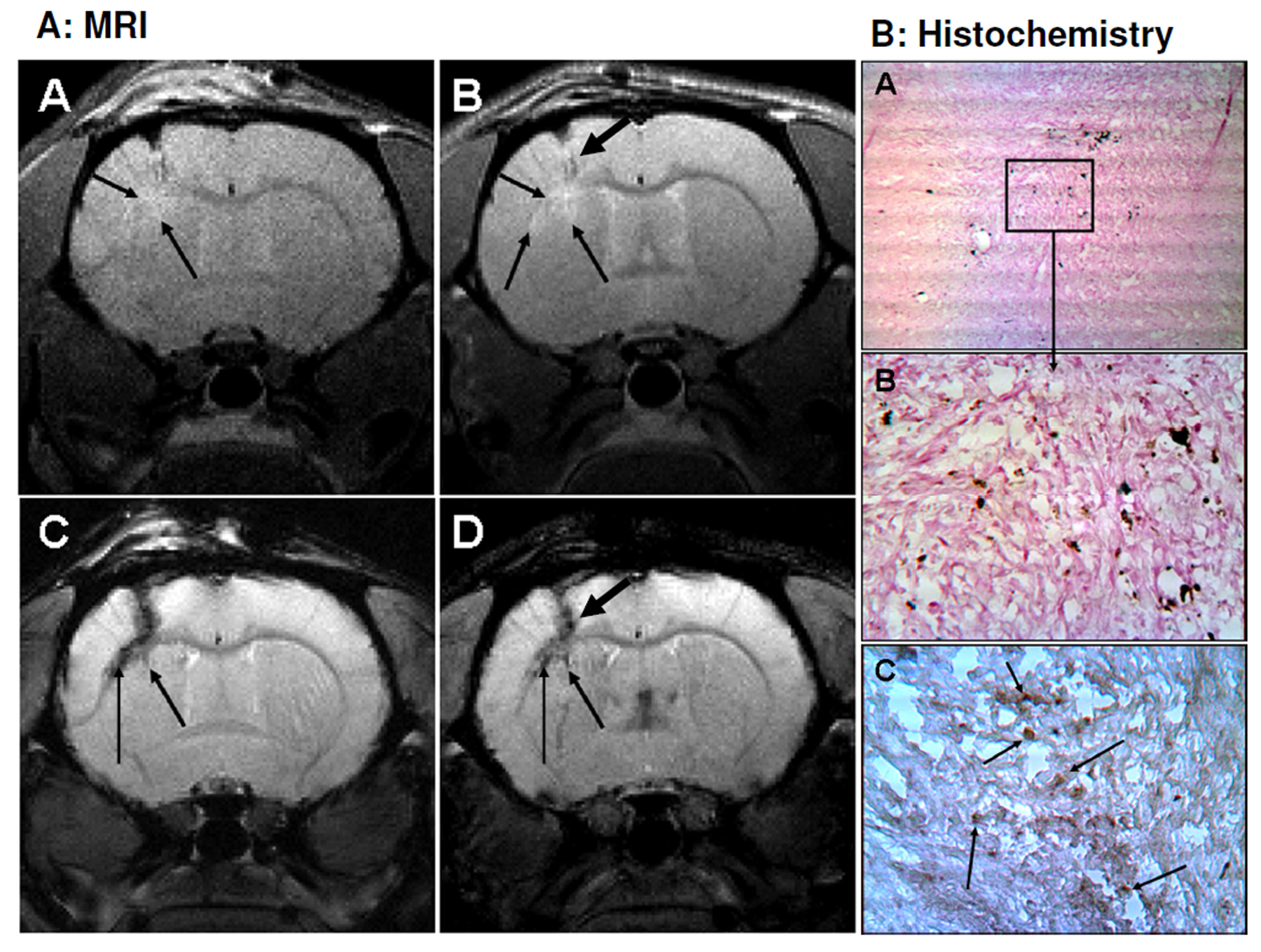

Supplement: Figure S5 — Detection of smaller U251 tumor. Magnetically labeled CTLs were injected intravenously in rats bearing U251 implanted tumor (n = 3) on day 7 after the implantation. Both T2-weighted (T2WI) and T2*-weighted (T2*WI) images were acquired on day 3 and 7 after IV administration of CTLs. Following the last MRI, animals were euthanized and their brains including tumors were collected after perfusion and prepared for histochemical analysis. Images from representative experiment demonstrate migration and accumulation of administered CTLs in the small tumor. Figure S5A: T2WI shows high signal intensity areas (arrows) at the site that are considered to be the growing tumor at day 10 (A) and day 14 (B) following the implantation of tumor (day 3 and 7 after IV administration of labeled CTLs). Note the extension of tumor along and below the corpus callosum. T2*WI shows low signal intensity at the sites thought to be growing tumor (arrows) at day 3 (C) and day 7 (D) after the administration of labeled CTLs. Note the low signal areas that are indeed within the high signal areas seen on T2WI. The low signal area seen in the cortex (thick arrow) might be due to needle track and hemorrhage during implantation of tumor. Figure S5B: DAB enhanced Prussian blue (A, B) and CD45RO (C) staining from consecutive sections showing the accumulated iron positive cells (dark brown) in the small tumor mass (A, 10x) and (B, 40X). CD45RO staining shows multiple activated T-cells within the tumor (arrows, Magnification 40X). (9.53 MB TIF) [file pone.0009365.s006.tif]
